# The effectiveness of an interactive audio‐tactile map for the process of cognitive mapping and recall among people with visual impairments

**DOI:** 10.1002/brb3.1650

**Published:** 2020-05-22

**Authors:** Edward Griffin, Lorenzo Picinali, Mark Scase

**Affiliations:** ^1^ School of Nursing and Midwifery De Montfort University Leicester UK; ^2^ Dyson School of Design Engineering Imperial College London London UK; ^3^ Division of Psychology De Montfort University Leicester UK

**Keywords:** assistive technology, audio‐tactile map, blind, visual impairment

## Abstract

**Background:**

People with visual impairments can experience numerous challenges navigating unfamiliar environments. Systems that operate as prenavigation tools can assist such individuals. This mixed‐methods study examined the effectiveness of an interactive audio‐tactile map tool on the process of cognitive mapping and recall, among people who were blind or had visual impairments. The tool was developed with the involvement of visually impaired individuals who additionally provided further feedback throughout this research.

**Methods:**

A mixed‐methods experimental design was employed. Fourteen participants were allocated to either an experimental group who were exposed to an audio‐tactile map, or a control group exposed to a verbally annotated tactile map. After five minutes’ exposure, multiple‐choice questions examined participants’ recall of the spatial and navigational content. Subsequent semi‐structured interviews were conducted to examine their views surrounding the study and the product.

**Results:**

The experimental condition had significantly better overall recall than the control group and higher average scores in all four areas examined by the questions. The interviews suggested that the interactive component offered individuals the freedom to learn the map in several ways and did not restrict them to a sequential and linear approach to learning.

**Conclusion:**

Assistive technology can reduce challenges faced by people with visual impairments, and the flexible learning approach offered by the audio‐tactile map may be of particular value. Future researchers and assistive technology developers may wish to explore this further.

## Graphical abstract

People with visual impairments can experience numerous challenges navigating unfamiliar environments. This mixed‐methods study examined the effectiveness of an interactive audio‐tactile map for the process of cognitive mapping and recall among people who were blind or had visual impairments. The audio‐tactile map condition had significantly better overall recall than the control group. The interviews suggested that the interactive component offered individuals the freedom to learn the map in several ways and did not restrict them to a sequential and linear approach to learning.

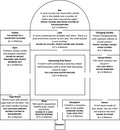

## 1 INTRODUCTION AND REVIEW OF PREVIOUS WORK

Cognitive components of thought and reasoning can resemble real‐world objects and the spatial aspects of environments (Kosslyn, 1880; Tversky & Bower, 1991). The gathering, encoding, storing, and retrieving of information for everyday navigational tasks are known as cognitive mapping (Campus et al., 2012). There has been considerable focus on the cognitive and neurological systems underpinning such processes (Cohen & Eichenbaum, 1991; Eden, 1988; Kupers, Chebat, Madsen, Paulson, & Ptito, 2010; O'Keefe & Nadel, 1978; Tolman, 1948). Cognitive maps consist of information about spatial relations and distances between objects (Bestgen & Dupont, 2003; Johnson‐Laird & Posner, 1989). They can also include representations of fictional places or of locations in their physical absence (e.g., the layout of a building). Physical maps can function as a prenavigation tool, which enables cognitive mapping of previously unknown environments (Ungar, Blades, & Spencer, 1993). This can help facilitate an understanding of the environment in terms of spatial awareness, orientation, and navigation (Ungar et al., 1993).

People with visual impairments can encounter multifarious challenges navigating unfamiliar indoor and outdoor environments (Strelow, 1985; Ungar et al., 1993). While familiar surroundings may also present problems, having previous experience or spatial knowledge can provide valuable assistance. In a review of literature, Cattaneo et al. (2008) identified similarities in how visually impaired individuals and those with normal vision processed visual information. In addition, individuals with congenital blindness (i.e., those having never experienced visual stimulation) had comparable experiences of mental imagery to people without visual impairments. These included the use of similar cognitive and, to a slightly lesser extent, neurological mechanisms. Furthermore, there are arguments suggesting that blind people can compensate for their visual deficit by utilizing other nonvisual forms of information. For example, congenitally blind individuals have been found to possess superior tactile acuity (Cattaneo et al., 2008; Goldreich & Kanics, 2006), better memory of voices, verbal material (Roder & Neville, 2003), and auditory localization (Roder et al., 1999). However, some studies have reported inferior sensory and cognitive processes among blind individuals (Gagnon, Kupers, & Ptito, 2013; Gori, Sandini, Martinoli, & Burr, 2014).

Assistive technology (e.g., tactile maps, auditory simulations, haptic navigation, global positioning systems) can stimulate cognitive mapping processes to provide navigational assistance for visually impaired individuals (Roentgen, Gelderblom, Soede, & de Witte, 2008), which can be used to support real‐time navigation (Ertan, Lee, Willets, Tan, & Pentland, 1998; Katz et al., 2012) and prenavigation (Jacobson & Kitchin, 2012; Katz & Picinali, 2011; Picinali, Afonso, Denis, & Katz, 2014). Maps with tactile components can be assimilated through technology to utilize the haptic perception system via the sense of touch (O'Sullivan, Picinali, Gerino, & Cawthorne, 2015). These can be useful prenavigational instruments, which can provide kinesthetic information about an object or environment (Campus et al., 2012), using embossed representations of geographic and route‐related features. Such instruments can facilitate navigation by providing survey and route‐related knowledge of an environment (Ungar et al., 1993), and can aid spatial learning, orientation, spatial choice, decision making (Jacobson & Kitchin, 2012), travel speed, safety, and confidence (Roentgen et al., 2008). Tactile typography such as Braille can provide information regarding key features of the map. Research has identified that annotated tactile maps for prenavigation, with the inclusion of a Braille display, were received positively by participants (Zeng & Weber, 2011). However, Braille and other tactile print usually require more space than printed text. For example, a single page of print can be 2.5–3 pages in length when translated into Braille. Thus, tactile maps are limited regarding the amount of Braille that can fit onto them, making it difficult to convey smaller and more detailed information such as gradient and individual road names.

More recently, both prenavigation and real‐time navigation systems for visually impaired people have benefitted from new technologies to facilitate greater interaction and engagement. These include video game prenavigation approaches (Marebet, Connors, Halko, & Sánchez, 2012), interactive hepatic maps on tablet/mobile devices, where the map vibrates to simulate a specific point of reference (i.e., a wall or object of interest; Papadopoulos, Koustriava, & Koukourikos, 2018), the use of Bluetooth, wireless, and GPS technology to provide participants with interactive real‐time feedback (Martinez‐Sala, Losilla, Sánchez‐Aarnoutse, & García‐Haro, 2015; Meliones & Sampson, 2018), and electronic devices with actuated pins. Research has generally implied that interactive approaches offer superior performance than conventional methods (Brayda, Leo, Baccelliere, Ferrari, & Vigini, 2018; Marebet et al., 2012; Martinez‐Sala et al., 2015; Meliones & Sampson, 2018) in terms of learning, navigation, orientation, and recall. For example, programmable array matrices with haptic feedback for prenavigation offer superior spatial performance to conventional procedures on static tactile maps (Brayda et al., 2018). The recognition of tactile feedback, even when applied to the feet, has shown to be of high accuracy among visually impaired individuals (Velázquez et al., 2018). However, some interactive systems have been criticized for being overly complicated, inaccurate, and difficult to use (Martinez‐Sala et al., 2015).

One relatively simple alternative to using Braille is a tactile map accompanied by audio annotation. Graf (2010) argued that tactile maps that only represent spatial information were less optimal for aspects associated with navigation and location than tactile maps accompanied by verbal annotation. Other research has found that replacing Braille on an interactive map with simple audio‐tactile interaction significantly improved efficiency and participant satisfaction among blind individuals (Brock, Truillet, Oriola, Picard, & Jouffrais, 2015). However, the effectiveness of the map was not significantly improved over a Braille system and more associated with participant capabilities. Interactive multimodal systems that combine auditory and tactile modalities have shown to be effective in nonvisual navigation (Geronazzo, Bedin, Brayda, Campus, & Avanzini, 2016).

O'Sullivan et al. (2015) integrated a paper tactile map, with an off‐the‐shelf computer tablet device. The audio‐tactile map (ATM) prototype was a tablet computer (9.7‐inch screen) overlain with a paper tactile map, printed onto specialized swell paper that was passed through a fuser oven to create raised textures. The tablet screen mirrored the paper map that was overlain, aligned, and attached to the screen. The tablet computer enabled the provision of touch‐activated audio feedback when the participant interacted with it. A partially sighted individual had significant involvement in the design and assessment stages, where qualitative and quantitative feedback was provided. This focused on the design of the map and the auditory and tactile feedback. The ATM prototype was developed as a prenavigation tool for learning a new environment before visiting it. It was devised to offer an interactive, multimodal experience that was informative, fun, and accessible. The ATM prototype offers individuals a more flexible approach to learning a new environment by providing various tactile and audio stimuli to facilitate learning. Papadopoulos et al. (2018) identified that similar audio‐tactile maps using tablet devices were more effective than a verbal description of a journey.

## 2 METHOD

The current research followed on from O'Sullivan et al. (2015) work, by examining the effectiveness of the ATM prototype on the process of cognitive mapping and recall, among people with visual impairments. When learning a new environment, people with visual impairments have been shown to prefer route or journey‐like approaches (egocentric) as opposed to survey‐like strategies (allocentric) that provide a more holistic overview of the area (Besse et al., 2017; Noordzij, Zuidhoek, & Postma, 2006). This may be because blind individuals acquire information about their environment serially (Leo et al., 2019). However, survey‐like approaches have been found to be superior among blind individuals (Chiesa, Schmidt, Tinti, & Cornoldi, 2017; Leo et al., 2019), but are harder to learn. Route‐like approaches to learning a journey are typically linear, but do not allow participants the flexibility to navigate their own route through the map. Therefore, the current study will compare an audio‐tactile map prototype, offering a flexible approach to learning an environment, with a conventional tactile map accompanied by a route‐based verbal description. We examined how accurately individuals could recall the environment represented in the map after five minutes’ exposure, by asking them a series of questions testing their memory of the map and its contents. In addition, participants were interviewed about their experiences of the study, the product, and how they thought it could be improved. Due to the audio‐tactile map multimodal qualities, which can allow for multiple learning styles, it was predicted that participants exposed to the audio‐tactile map would answer more questions correctly than those using a tactile map accompanied by a verbal description.

### 2.1 Participants

**FIGURE 1:**
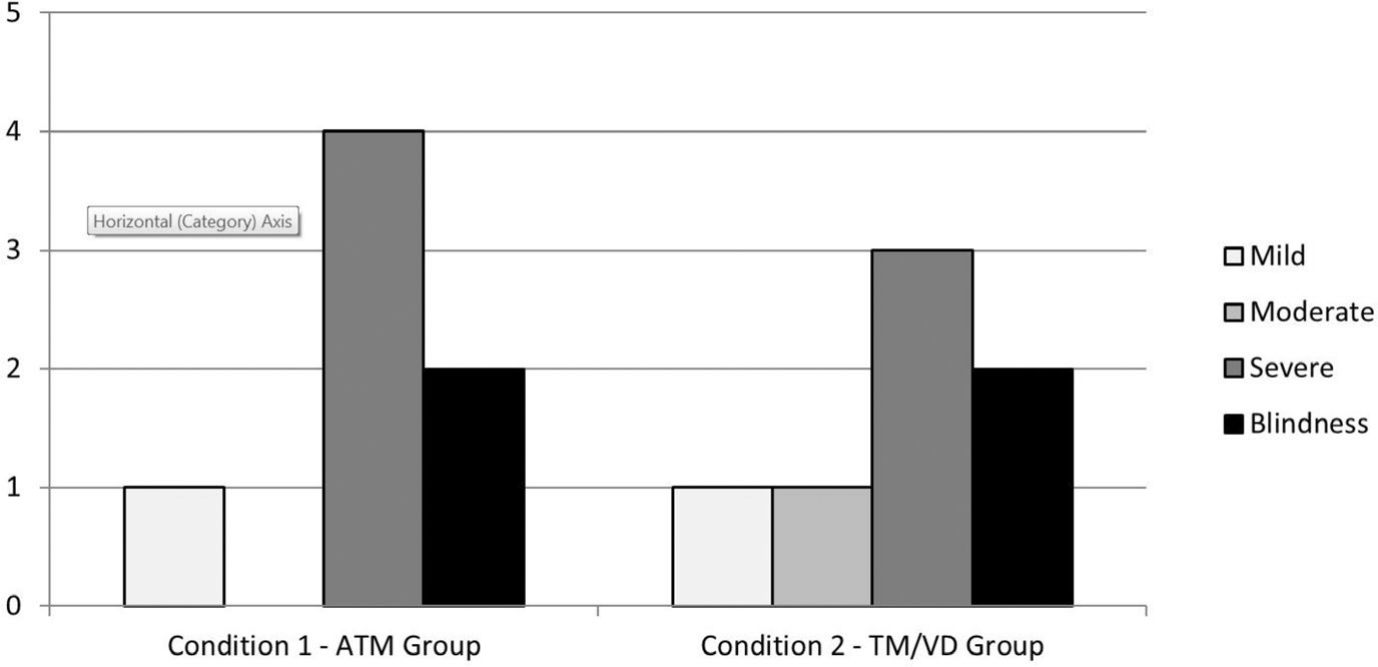
Bar chart showing the numbers of individuals in each group reporting “mild,” “moderate,” or “severe” levels of visual impairment, or “blindness”

Fourteen volunteers (eight male and six female) with visual impairments took part in both the quantitative (Phase 1) and subsequent qualitative (Phase 2) components of the study. All participants reported visual impairments ranging from mild (i.e., vision assisted by lenses, but not entirely corrected) to complete blindness (i.e., no vision at all). Further details about their specific impairments were not formally acquired, but those reporting diagnosed memory impairments were excluded. Ages ranged from 30 to 65 years (*M* = 48.8; *SD* = 14.4), with two participants not disclosing this information. All volunteers were randomly assigned to either an experimental group (Condition 1) or a control group (Condition 2) using a matched‐pairs approach. Too frequently in visual impairment research, participants are classified by their formal medical deficits, irrespective of their endorsement of such labels. Factors such as learning experience and social environmental aspects may be more relevant to performance than classic diagnostic distinctions (Loomis et al., 1993). Therefore, participants were matched based on their self‐reported level of severity regarding their visual impairment (e.g., mild, moderate, severe, blind). However, due to a slightly uneven distribution, the experimental group had a marginally higher visual impairment level (see Figure 1). Participants consisted of university staff (*n* = 3), members of the Vista Blind organization (*n* = 10; www.vistablind.org.uk), and individuals previously known to the research team (*n* = 1). In addition to research involvement, participants were asked about their experiences of the product and how they thought the prototype could be improved.

### 2.2 Theoretical background and design

This research employed a mixed‐methods approach, combining quantitative and qualitative procedures. A sequential QUAN → QUAL mixed‐methods design was used (Teddlie & Tashakkori, 2009), whereby a quantitative experimental study (Phase 1) preceded semi‐structured interviews with the same participants (Phase 2). The quantitative component informed the qualitative strand in terms of providing a focal point for discussion, while the qualitative elements helped explain potential factors contributing to the performance between groups.

#### 2.2.1 Phase 1: Quantitative

The first phase of this research employed a between‐groups experimental design, comparing the effectiveness of two map conditions (independent variable), on the process of cognitive mapping and recall of the map. The experimental group (Condition 1) was exposed to an interactive audio‐tactile map for 5 min, while the control group (Condition 2) was exposed to an identical noninteractive tactile map accompanied by a verbal description of a journey through that map for the same amount of time. After exposure to one of the map conditions (followed by a 1‐min break), participants completed a series of 20 multiple‐choice questions assessing orientation and spatial awareness (dependent variable). Both map conditions were based upon the same fictional building of a health club, and the information that was provided to participants in both conditions reflected the same aspects of the map. However, the interface in which participants received the information differed.

#### 2.2.2 Phase 2: Qualitative

The second phase involved the collection and analysis of qualitative data via semi‐structured interviews. Participants from both groups were invited to experience the audio‐tactile map and asked a series of questions about the experiment and about their views and feedback regarding the prototype. Data ascertained were analyzed using thematic principles (Braun & Clarke, 2013). Detailed notes were taken by the researcher and were analyzed in the qualitative software package NVivo, by employing the following steps: (a) Data familiarization—writing and rereading notes; (b) Coding—line‐by‐line coding and categorizing of notes, (c) Searching for themes—linking the codes into meaning themes, (d) Reviewing the themes—Checking the evidence for the themes and amending if needed, (e) Defining and naming themes, and (f) Writing up analysis (Braun & Clarke, 2013).

### 2.3 Materials

**FIGURE 2:**
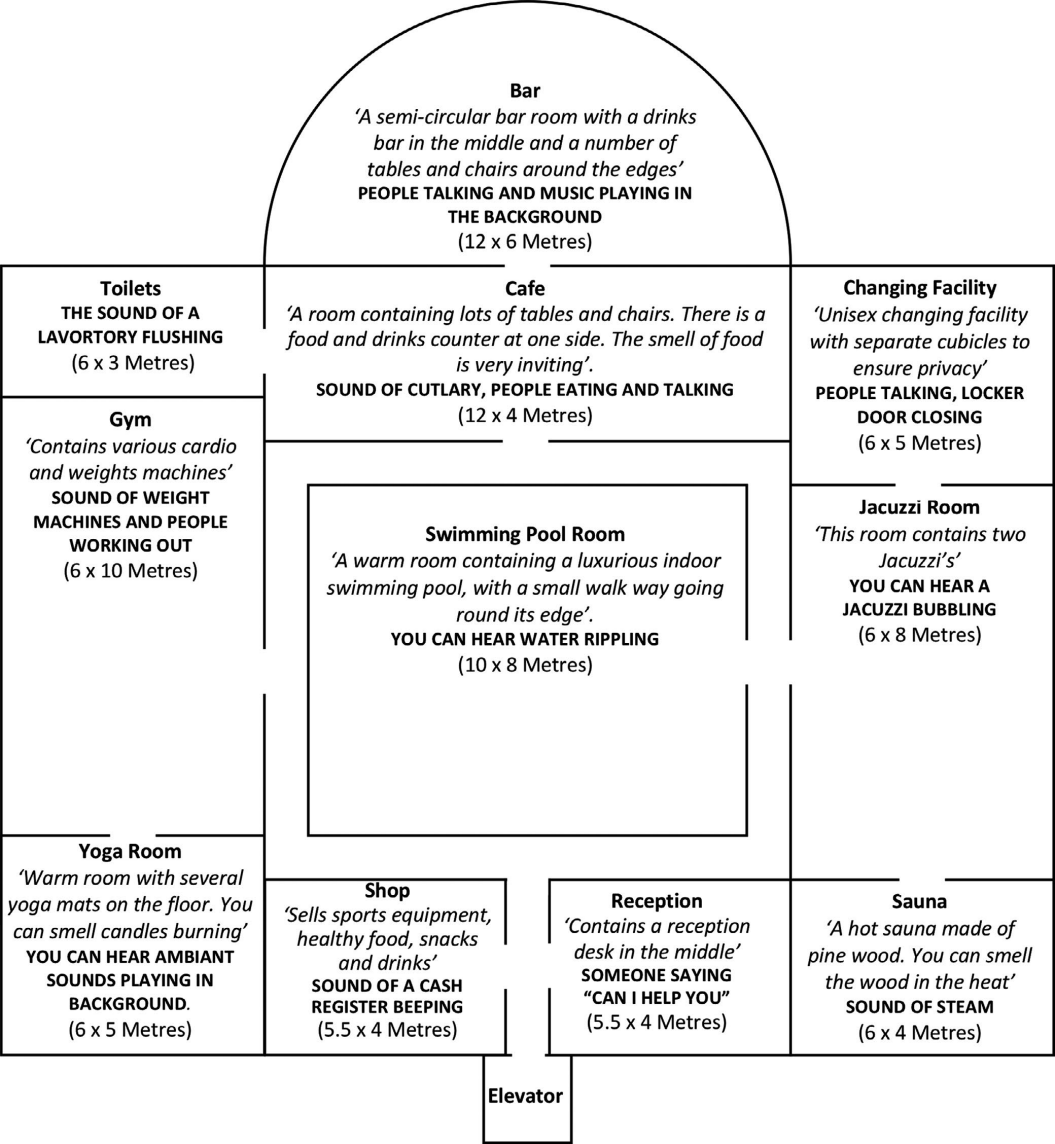
A representation of the map developed for this experiment, including a description of the rooms and sound effects

A fictitious map depicting a health club (see Figure 2) was used for both conditions because of its distinctive rooms (e.g., Swimming Pool Room, Gym, Café, Sauna) with unique characteristics regarding atmosphere, sound, and other sensory stimuli. The map was incorporated into an audio‐tactile map (Condition 1) and a conventional tactile map with an accompanying description (Condition 2). To control for potential confounding variables, both maps provided the same detail and information. Two map designs were initially developed for this research, and their advantages and disadvantages were discussed among the research team, with consideration of accessibility to people with visual impairments. As discussed above, an individual with visual impairments had a key role in the development of the ATM system and part of this analysis included further feedback surrounding this system to be utilized for further development.

#### 2.3.1 Condition 1: ATM

**FIGURE 3:**
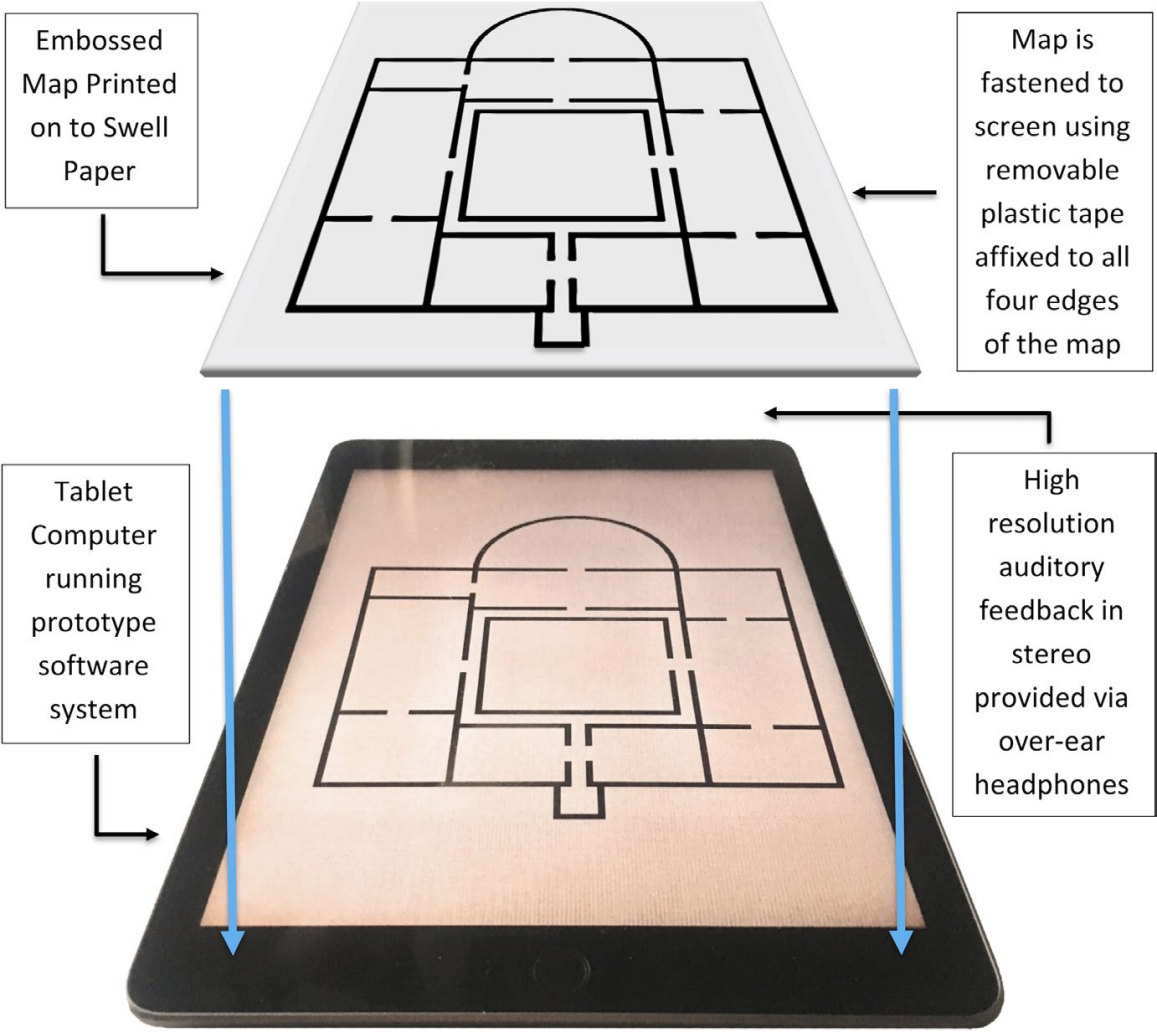
A diagram showing the key operational components of the audio‐tactile map prototype

This prototype included a tactile map printed onto swell paper where the internal and external walls and door spaces were embossed. This was attached to tablet computer screen (9.5ʺ × 7.3ʺ) using removable plastic tape. The prototype software displayed a static image of the map with the same dimension as the embossed paper map, which was aligned to the image. The software included background sound, audio description, and acoustic‐click feedback (described below) to reflect the room size and acoustics (see Figure 3). Participants could interact with the audio‐tactile map in three ways: (a) Moving the finger inside a specific room activated its corresponding background noises (bold text; see Figure 2); (b) Tapping twice inside the room activated the playback of text‐to‐speech auditory information about the specific room (italic text; see Figure 2); and (c) Tapping three times inside the room activated the acoustic‐click feedback, which emulated how a finger click would sound in each space, including factors such as echo, reverberation, and the acoustic properties of each room.

For the first and the third interactions, the audio signals (background and finger clicking noises) were rendered through virtual acoustic simulations, in order to emulate how they would have sounded in the various rooms. All spaces were precisely modeled using acoustic prediction and auralization software (CATT‐Acoustic—http://catt.se/). For an in‐depth technical discussion of the ATM prototype, please see O'Sullivan et al. (2015). Data such as room size, acoustic property of materials on the walls, ceiling and floor, and various objects in the rooms (e.g., sofas, desks) were accurately modeled. The noises were then reproduced in the virtual environments, in order to simulate the appropriate echo feedback for the room acoustical characteristics. The audio was optimized for headphone playback using the binaural technique, in order to give a strong sense of immersion in the virtual environment (O'Sullivan et al., 2015). To give an example of the effects of such processing, the large swimming pool room generated an echo with a longer delay than the smaller sauna area, to denote its size. It also had a higher level of reverberation than the other rooms, to simulate the effects that the hard and reflective surfaces typically found in a swimming pool area. Conversely, the small shop area had a comparatively short delay and less reverberation due to containing numerous objects (i.e., merchandize, till area).

#### 2.3.2 Condition 2: Tactile map with verbal description

This condition involved five minutes’ exposure to a noninteractive paper tactile map of the same fictional health club. The researcher narrated a verbal description of a journey through the map and included a description of the shape and size of the room, the background sounds, and the objects within the room (see Appendix 1 for full description). The sequential journey took participants through all of the rooms. After a brief familiarization with the map, participants were required to start at the elevator and trace their journey using the tactile map. While the journey was sequential, participants were allowed to have details repeated as many times as required within the allotted time frame.

A series of 20 multiple‐choice questions were developed to examine the recollection and knowledge of the fictional environment. Five groups of four questions assessed different aspects of orientation and spatial awareness. Each of group contained questions of varying cognitive difficulty.

Four questions assessed aligned directional awareness using cardinal points (north, east, south, west):

1. You are standing in the elevator facing north. In what direction is the swimming pool room?
2. You are standing in the Reception facing north. In what direction is the Shop?
3. You are standing in the Sauna facing north. In what direction is the Yoga Room?
4. You are standing in bar facing north. In what direction is the Elevator?

Four questions assessed aligned directional awareness using ordinal points (northeast, northwest, southeast, southwest):

- You are standing in the center of the Gym facing north. In what direction is the Cafe?
- You are standing in the sauna facing north. In what direction are the toilets?
- You are standing in the Changing Facility facing north. In what direction is the Yoga Room?
- You are standing in the Toilets facing north. In what direction is the Swimming Pool Room?

Four questions assessed misaligned directional awareness using subjective orientation (in front, behind, to your left, to your right):

- You are standing in the center of the Cafe facing south. In what direction is the Swimming Pool Room?
- You are in the swimming pool room facing east. In what direction is the Bar?
- You are standing in the center of the shop facing west. In what direction is the Sauna?
- You are standing in the center of the Jacuzzi Room facing south. In what direction is the Gym?

Four questions examined map memory by asking participants about the fewest number of doors they would need to travel through to get from one room to another (one room, Two rooms, Three rooms, Four rooms).

- You are in the Cafe and you want to get to the Bar. What is the smallest number of doors that you would need to travel through?
- You are in the Reception and you want to get to the Swimming Pool Room. What is the smallest number of doors that you would need to travel through?
- You are in the Changing Facility and you want to get to the Sauna. What is the smallest number of doors that you would need to travel through?
- You are in the Yoga Room and you want to get to the Changing Facility. What is the smallest number of doors that you would need to travel through?

Four questions examined room size by asking participants to identify the largest or smallest out of two rooms. For example: Which room is the largest Room? (Gym or Jacuzzi Room).

- Which is the largest Room? (Shop or Swimming pool Room)
- Which is the smallest Room? (Sauna or Yoga Room)
- Which room is the largest Room? (Gym or Jacuzzi Room)
- Which room is the smallest Room? (Yoga Room or Shop)

#### 2.3.3 Procedure and analysis

Phase 1: Ethical approval was granted by the faculty ethics committee at De Montfort University. Firstly, participants provided demographic details concerning their sex, age, level, and type of visual impairment and whether they had a diagnosed memory impairment. After five minutes’ exposure to one of the map conditions (followed by a 1‐min break), participants completed the 20 multiple‐choice questions, for which scores between the conditions were compared. Scoring was binary as participants scored 1 point for each correct answer and 0 points for incorrect answers. As the questions had the potential effect of contributing to the participants learning of the map, it was considered more beneficial to ask the questions in the same order to all participants. After completing the questions and undergoing a debriefing, participants were invited to explore the audio‐tactile map prototype prior to Phase 2 of the study.

Quantitative data were analyzed using nonparametric procedures via the statistical software package SPSS. These included the Mann–Whitney *U* tests comparing between‐groups performance and Spearman's rank correlation for preliminary descriptive analysis. Nonparametric tests are not based on the mean, variance, and probability distributions of scores, but instead focus on their ranked order and sum of ranks (Field, 2018). Significance is generally determined by the mean rank and standard error (Field, 2018). Thus, nonparametric tests make minimal assumptions surrounding normality and are less susceptible to deviations and outliers (Kraska‐Miller, 2014). Therefore, they are more suitable for smaller samples, where normality of the distributions is difficult to determine. Alongside the main between‐groups analysis of the overall test scores, further comparisons using the Mann–Whitney *U* tests are reported comparing the between‐groups scores for the four individual subsections of the test. These subsections included the following: Aligned Orientation (eight questions assessing orientation in relation to cardinal and ordinal points), Misaligned Orientation (four questions assessing directional awareness relative to subjective orientation), Shortest Journey (four questions regarding to the shortest journey between rooms), and Room Size (four questions comparing the size of two rooms).

Phase 2: After completing Phase 1 of the study and spending some time experiencing the audio‐tactile map prototype, participants were asked a series of five questions: (1) “How did you find the study?” (2) “What are your views surrounding the audio‐tactile map?” (3) “To what extent could the audio‐tactile map help you learn a new environment?” (4) “What are the strengths and weaknesses of the audio‐tactile map and how does it compare to other approaches that you have experienced?” (5) “How could this item be improved to assist you more effectively?” To maintain a positive and relaxed participant experience, it was chosen not to record the interview. Detailed researcher notes were therefore an integral part of the data collection process. The qualitative software package NVivo 10 was utilized to assist with data analysis. The data were imported into the program, and a thematic analysis was conducted. The six steps of analysis (see Section 2.2) suggested by Braun and Clarke (2013)  were applied, using the node and tree‐node functions. The text search facility was utilized in Step 4 to speed up the process of reviewing themes.

### 2.4 Ethical statement

Ethical approval was granted by the faculty ethics committee at De Montfort University.

## 3 RESULTS

### 3.1 Phase 1: Quantitative analysis

#### 3.1.1 Descriptive analysis

The mean age for males (*n* = 8) was 52.1 years (*SD* = 12.1) and females (*n* = 6) was 44.0 years (*SD* = 17.4). The mean age of participants in Condition 1 was 51.7 years (*SD* = 14.0), while that of those in Condition 2 was 44.6 years (*SD* = 15.5). However, there were no significant correlations between age and total test score for Condition 1 (*r*_s_ = .064, *N* = 7, *p *= .89) and Condition 2 (*r*_s_ = .027, *N* = 5, *p *= .97), suggesting that age was not related to performance in either of the conditions. In addition, there were no significant differences in overall scores between participants who reported early blindness (*Md* = 14, *n* = 6) and late blindness (*Md* = 14.5, *n* = 6), *U* = 14.000, *z* = −645, *p *= .589. Finally, there was no significant relationship between level of visual impairment (*1 = mild, 2 = moderate; 3 = severe; 4 = blindness*) and overall performance (*r* ^s^ = .26, *N* = 14, *p *= .42).

#### 3.1.2 Between‐groups analysis

**FIGURE 4:**
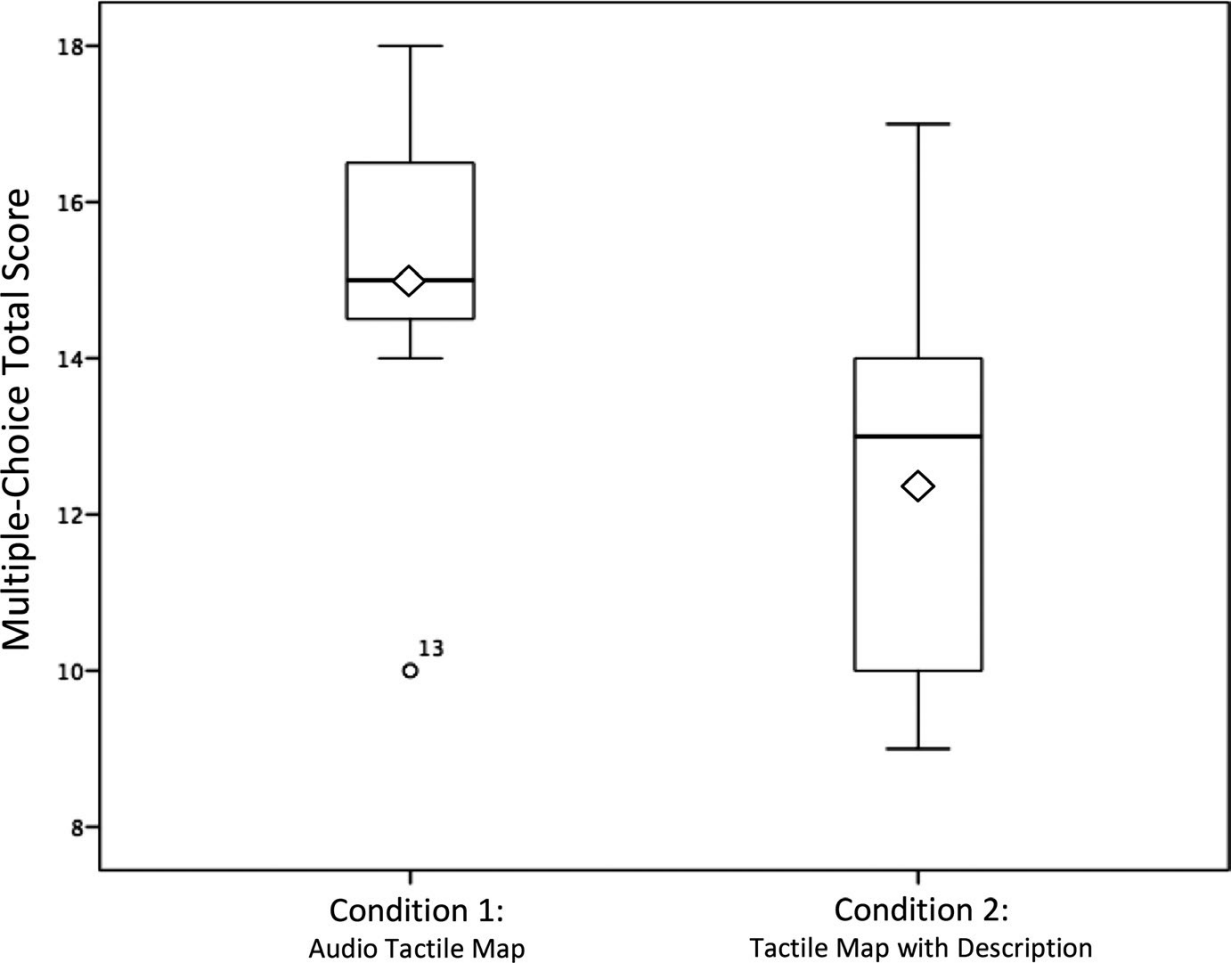
A box plot displaying the data distribution (range, upper and lower quartiles, and median) and mean (represented by the diamonds) of the overall scores on the multiple‐choice test for both conditions

A Mann–Whitney *U* test identified that the overall scores for the 20 multiple‐choice questions were significantly higher for Condition 1 (*Md* = 15, *n* = 7) than Condition 2 (*Md* = 13, *n* = 7), *U* = 11.500, *z* = −1.68, *p *= .042 (one‐tailed), *r *= .45, indicating a medium to large effect size using Cohen's 1988 criteria (i.e., 0.3 = medium, 0.5 = large; please see Figure 4 and Table 1 for details). The Condition 1 median score for males (*n* = 5) was 15.0 (mean = 14.8, *SD* = 2.95) and that for females (*n* = 2) was 15.5 (mean = 15.5, *SD* = 2.12). The Condition 2 median score for males (*n* = 3) was 15 (mean = 14, *SD* = 3.61) and that for females (*n* = 4) was 11.5 (mean = 11.3, *SD* = 2.06).

**TABLE 1:** Means, medians, and standard deviations for the overall test scores and the subsections between the two groups

	Condition 1 (*n* = 7) Audio‐tactile map			Condition 2 (*n* = 7) Annotated tactile map			*U*	*z*	*p *(one‐tailed)	*r*
	Mean	*SD*	Median	Mean	*SD*	Median				
Overall score	15.00	2.58	15.00	12.43	2.94	13.00	11.50	−1.68	.042	.45
Subsections										
Aligned orientation	6.00	1.53	6.00	4.86	1.07	5.00	13.00	−1.50	.066	.40
Misaligned orientation	2.57	1.13	2.00	2.29	1.11	2.00	21.00	−0.46	.322	.12
Shortest journey	2.86	1.07	3.00	2.29	0.95	3.00	16.50	−1.10	.136	.29
Room size	3.57	1.13	4.00	3.00	0.82	4.00	13.00	−1.64	.051	.44

When the five groups of questions were analyzed separately, individuals in Condition 1 typically performed better. However, none of these individual analyses achieved significance. However, it should be noted that differences in questions examining aligned orientation and room size were nearing significance (Table 1).

### 3.2 Phase 2: Qualitative analysis

While Phase 1 provided evidence supporting the audio‐tactile map's effectiveness as a prenavigation tool, it was considered pertinent to examine participants’ views of the prototype and the study in general. Participants from both conditions were provided an opportunity to explore the audio‐tactile map after phase 1 of the study had been completed. They provided multiple discourses and narratives to communicate their views and experiences. While each individual contributed a unique perspective, several similarities emerged surrounding their attitudes toward the audio‐tactile map. In addition, participants also discussed the challenges associated with visual impairment and their experiences of assistive technology. Nearly all participants used language to suggest the audio‐tactile map would be useful and beneficial to them. Focusing specifically on the research questions, three themes emerged from the data:

#### 3.2.1 Theme 1: The value of flexible learning

Some participants articulated praise toward the audio‐tactile map's multiple components of navigational assistance. A combination of audio and tactile stimuli delivered in a nonsequential way appeared to create perceptions of choice and freedom. These multimodal elements provided additional dimensions to the annotated description in Condition 2. Participants from both Conditions reflected positively about the combination of stimuli available with the ATM.

I like this one better because you’ve got lots of different ways of learning about the rooms (Condition 2 : Male)

There was a general feeling that the audio‐tactile map condition was less restrictive and provided more freedom than the sequential journey route offered in Condition 2.

“The first one gives you a path, but this one, you can choose your own” “If you forget where you’ve been, you can just go back” (Condition 1 : Male)

The audio‐tactile map offered a system where a single touch of the room activated a sound effect, which characterized the typical sounds present in that space. For example, by touching on the cafe room the sounds of diners talking and using cutlery could be heard. Some participants emphasized that they found the sound effects particularly useful in helping to install a memory of the space. Having three levels of audio stimuli appeared to contribute to this view of flexible learning.

It is much better having the sound effects than having someone just reading a description. (Condition 1 : Male)

#### 3.2.2 Theme 2: An intuitive and fun approach to learning

With new technology enabling more authentic replications of environments, the developers of the audio‐tactile map prototype endeavored to provide realistic simulations of sound for this virtual map. Six participants used language suggesting they found the audio‐tactile map “easy” to use and “accessible.” One participant commented on how intuitive she found the system:

It is good fun, and it is easy to use. Having the sounds play makes it feel real and it results in it feeling very intuitive. (Condition 1 : Female)

The same participant further added:

We’re used to tablet PCs, so having something that uses the features makes it nice to use. I don’t feel like I have to relearn much. (Condition 1 : Female)

The audio‐tactile map offered additional layers of interaction not present in Condition 2, which consisted of a tactile map accompanied by a journey‐based verbal description. However, both of these conditions conveyed the same details, albeit using different formats. When asked for their views surrounding the audio‐tactile map, participants typically reflected positively about the fun factor that the interface provided, particularly when compared to Condition 2:

This is much more fun to use than the other one. The sounds make it more enjoyable (Condition 2 : Male)

One participant from Condition 1 seemed particularly enthusiastic about exploring the audio‐tactile map prototype. He was reluctant to pass it back to the researcher prior to the interview. He commented that he “really liked it” and that it was “good fun” and “entertaining.” Participants also reflected positively about the audio description that was played when they double tapped their finger on each specific room. They appeared to rely heavily on this aspect with regard to learning the building.

#### 3.2.3 Theme 3: Recommendations for developing and improving the audio‐tactile map

Some participants raised concerns that their “voices” were “not always heard” in assistive technology development circles, particularly regarding the products that they used. Several suggestions were made about improving the audio‐tactile map prototype and how such approaches would be useful. While the audio‐tactile map prototype was designed as a prenavigation tool, two participants asked whether it was compatible with global positioning system (GPS) technology. One of whom felt that a GPS could be “really useful” in allowing individuals to take the audio‐tactile map with them to a new location and identify where they were within the map.

The audio‐tactile map prototype used for the study had the paper tactile map affixed to the screen using tape, but designs for a more accessible system were under discussion with the research team. One individual recommended a sliding mechanism for affixing the tactile map to the tablet computer screen. He suggested a “clip‐on” cover might be useful for aligning the map with the screen.

It was also suggested that objects of interest within the rooms could be marked on the map for which an additional description could be activated by an alternative tap gesture. Participants appeared to have preferences for one or more of the three sound effect options activated by clicking the screen. For example, several chose to rely more on the audio description but found the sound effects a little “annoying” when accidently activating them during map exploration. Having a more flexible system so that individuals could choose how to activate the sounds may be of value. This taps into the concept of flexibility and having a system that accommodates the various and specific needs of each individual.

## 4 DISCUSSION

Visually impaired and blind individuals can experience numerous challenges when navigating unfamiliar environments (Ungar et al., 1993). Tactile maps can be of value to such individuals (O'Sullivan et al., 2015; Zeng & Weber, 2011), particularly when accompanied by verbal or audio feedback (Brock et al., 2015; Graf, 2010; Papadopoulos et al., 2018). The current research compared an audio‐tactile map with a verbally annotated tactile map (Graf, 2010) utilizing a sequential journey format (Noordzij et al., 2006), on cognitive mapping and recall. People using the audio‐tactile map had a significantly higher overall score on the 20 multiple‐choice questions designed to evaluate their recollection and cognitive mapping of the fictitious environment. Cognitive mapping and recall are key aspects of successful prenavigation, suggesting that the audio‐tactile map may be a more effective system than the tactile map accompanied by a verbal description. Noordzij et al. (2006) identified that a journey (egocentric) approach to learning an environment was preferable and more effective to survey‐based approaches among visually impaired individuals. Indeed, conventional tactile maps with an audio or verbal description (like the one used in Condition 2) offer such a method. However, a key restriction of such an approach is that the learning was linear and in a fixed sequence; thus, it did not allow participants the flexibility to navigate their own route through the map. The audio‐tactile map system, however, offered participants a flexible way of learning an environment, which allowed for both journey and survey strategies. While it was observed that the majority of participants appeared to employ both of these strategies (albeit to a greater extent in the ATM condition), further research would be required to confirm this.

The Condition 2 map provided information about the same aspects of the fictitious environment (i.e., background sounds and room size) provided in Condition 1, but the modalities for which the information was presented differed between the conditions. Instead of having actual sound effects, the sounds in the control condition were described as part of the verbal annotation read to the participants. The audio‐tactile map condition provided a more multimodal approach to learning, and some of the more lengthily description (i.e., sounds associated with the room and room sizes) were presented using sound effects as opposed to description. The use and engagement with the sound effects differed between participants, so it is difficult to determine whether they contributed to the superior performance in Condition 1. Previous research has suggested that blind individuals have better memory of voices and verbal material (Gori et al., 2014) and auditory localization (Gagnon et al., 2013). Therefore, this may have combined with the flexible approach to learning offered by the audio‐tactile map system to contribute to a more detailed cognitive map.

Due to logistical constraints in recruiting large numbers of participants, the sample size for this research was relatively small. While there was a significant overall effect, the four individual subsections did not achieve significance when examined separately. However, “aligned orientation” and “room size” were nearing significance and, in light of the medium/large effect sizes, may have achieved this on a larger sample. Thus, replication of this study on a larger group would be of value for future research endeavors. While the current study examined the learning, cognitive mapping, and recall of a fictitious environment represented in a map, further evaluative research examining its effectiveness in subsequent navigation of an actual or simulated environment would be of particular value.

The qualitative feedback was typically positive and included some useful recommendations for improving the system, which could be incorporated into further development (an example of further ATM prototypes can be found in this video—https://imperialcollegelondon.app.box.com/s/reo3p0qtcqsjq4sgyp9oodda71wwblyq). To improve the robustness of future qualitative findings, researchers may benefit from employing one or more other researchers to code a sample of the data and then the degree of coder agreement be examined.

A degree of caution should be taken when drawing conclusions as to which components of the ATM facilitated better recall. We acknowledge that a series of experimental studies for which singular variables were tested independently would be optimal in identifying what aspects of the map were most effective (i.e., allocentric Vs egocentric, or the way in which the information was presented), but our focus was to compare the ATM prototype as a whole with a more conventional approach. The multiple approaches to learning an environment offered by the audio‐tactile map appeared to positively accommodate the diverse learning needs of individuals. While the sequential journey approach (such as the method required to learn the map in Condition 2) has been found to be effective in prenavigation (Chiesa et al., 2017; Noordzij et al., 2006), the flexibility to learn an environment may have been comparably important in the current study. Indeed, this may have been a key factor in the better recall experienced in Condition 1. Furthermore, the “fun and intuitive” aspects of the audio‐tactile map may have increased engagement among participants. The feedback from Vista Blind was generally positive, and they felt the product would be a useful tool for many of their members. The tools and techniques used to simulate the various acoustic environments and generate the spatialized signals are relatively complex and costly. However, more widely accessible tools are becoming available, which can facilitate nonexpert users to perform similar tasks, albeit at lower resolution and quality. Future researchers may wish to explore the impact of high‐quality and high‐resolution auditory simulations, and ultimately develop an integrated package for the creation of audio‐tactile maps.

In summary, the audio‐tactile map system, which allows for flexible multimodal learning, yielded superior performance to an annotated tactile map in the encoding and retrieval components of cognitive mapping. This evidence suggests that this system yields superior recall and may function as an effective prenavigation tool among individuals with visual impairments. O'Sullivan et al. (2015) reported that the experiences of participants using a similar audio‐tactile map system were typically positive. Generally, they engaged with the system and found it intuitive, easy to use, and fun. The provision of assistive technology has enabled people with disabilities to be less challenged by their environment, and the involvement of visually impaired individuals through the development process has highly improved the usability of the prototype. Considering that such approaches are continually being improved, the flexible learning approach offered by the audio‐tactile map may be a valuable addition to future assistive technology developers.

## 5 CONCLUSION

This mixed‐methods experimental study examined the effectiveness of and interactive audio‐tactile map (ATM) in terms of cognitive mapping and recall of spatial and navigational content. The interactive ATM demonstrated superior performance to a verbally annotated tactile among people with visual impairments. The multimodal, flexible learning approaches provided by the ATM appeared preferential to the sequential journey format offered in the annotated tactile map condition. Future researchers and assistive technology developers might benefit from exploring this concept further, alongside the effectiveness of an ATM as a prenavigation tool. Reducing the challenges associated with navigating unfamiliar environments is a important endeavor for supporting individuals with visual impairments.

## CONFLICT OF INTEREST

The authors declare no potential conflicts of interest with respect to the research, authorship, and/or publication of this article.

## AUTHOR CONTRIBUTION

All authors meet the authorship criteria as specified by Brain and Behaviour Journal. Edward Griffin: Conceptualization and design (equal); methodology (equal); audio‐tactile map design (equal); data collection and analysis (lead); writing original draft (lead); review and editing manuscript (lead). Lorenzo Picinali: Conceptualization and design (equal); methodology (equal); hardware and software development (lead), audio‐tactile map design (equal); data collection and analysis (significant contribution); writing original draft (significant contribution); review and editing manuscript (significant contribution). Mark Scase: Conceptualization and design (equal); methodology (equal); map design (equal); data collection and analysis (significant contribution); writing original draft (significant contribution); review and editing manuscript (significant contribution).

## Data Availability

Research data are not shared.

## Appendix 1

### VERBAL DESCRIPTION READ TO PARTICIPANTS FOR CONDITION 2

#### Stage 1

You are in the elevator of a tall building, travelling upwards. You hear a ding sound as the elevator stops and the door in front of you opens. You step out into the beginning of corridor. You are facing north.

#### Stage 2

You walk a couple of metres up the 1 m wide corridor and notice a door to your right and a door to your left. The door on your right leads to a square 4.5 × 4 metre reception room with a reception desk in the middle (THE PERSON SITTING AT THE DESK ASKS “CAN I HELP YOU?”). The door on your left leads to another square 4.5 × 4 m room which turns out to be a small shop selling sports equipment, health food snacks and drinks (YOU HEAR THE SOUND OF A TILL BEEPING). After looking in each room you continue north up the corridor for another couple of metres.

#### Stage 3

The corridor connects to the mid‐point of another corridor (12 m long) running from east to west. You turn right and follow the corridor to the east. At the end, you turn 90 degrees left into another corridor going north (10 m). At the mid‐point of this corridor are doors in both the left‐ and right‐hand walls.

#### Stage 4

You take the door on your left and enter a large 10 × 8 m room containing a rectangular swimming pool and a walkway running around its edge. It is nice and warm in this room and (YOU CAN HEAR THE SOUND OF WATER RIPPLING). You leave this room by the door in which you entered it.

#### Stage 5

Standing back in the corridor, you take the other door which is opposite to the door to the swimming pool room. You enter into a 6 × 8 m room containing two Jacuzzi's (YOU CAN HEAR THE SOUND OF THEM BUBBLING AWAY). The walls on your left and right each have a door in the centre. You take the door on your left.

#### Stage 6

Walking through the door you enter a 6 × 5 m uni‐sex changing rooms with separate cubicles to ensure privacy. After looking around, you go back into the Jacuzzi room and take the door other door on the wall opposite. You step into a hot sauna and smell the pine wood in the heat. The room is 6 × 4 m in size.

#### Stage 7

You retrace your steps through the Jacuzzi room and through the door and back into the corridor. You turn right and walk north to the end of the corridor. You turn left in to an adjacent corridor leading you west. The corridor is 12 m long. At the mid‐point of this corridor is a door on your right.

#### Stage 8

You walk through the door and enter a large cafe room measuring 12 × 4 m. It is full of tables and chairs with a counter at one side (YOU CAN HEAR THE SOUND OF CUTLERY AND PEOPLE CHATTING AS THEY EAT). The smell of food is very inviting. The wall in front of you has a door in the middle. You walk through the door.

#### Stage 9

You are now on a semi‐circular bar room of the same width as the previous room. There is some music playing in the background. There is a drinks bar in the middle of the room with a number of tables and chairs scattered around the edges. You go back into the cafe. To your right there is another door marked as a toilet which you visit before you leave (YOU HEAR THE SOUND OF A TOILET FLUSHING). You leave the cafe through the door in which you entered.

#### Stage 10

You are now standing in the corridor. You turn right and continue walking west to the end. You are now at the start of another corridor going south. Half way down this corridor is a door on your right.

#### Stage 11

You go through the door into a 6 × 10 m gym room. It contains various Cardio and weights machines (YOU CAN HEAR THE MACHINGS AND PEOPLE WORKING OUT). This room appears to be on the opposite side of the building to the Jacuzzi room. You notice a door in the middle of the south wall of this room.

#### Stage 12

You walk through this door into a warm 6 × 5 m yoga room. You can smell candles burning and notice there are several yoga mats on the floor. (YOU HEAR SOME AMBIENT SOUNDS PLAYING IN THE BACKGROUND) and sense this room is peaceful and tranquil. You turn around and retrace your steps back through the gym and leave the gym through the door in which you entered from the corridor.

#### Stage 13

You carry on walking south down the corridor. After about 5 m, you turn left into another corridor going east. You continue walking east to the mid‐point of the corridor. You turn right into the corridor you started in. You walk past the shop and the reception and back to the elevator.

## References

[brb31650-bib-0001] Besse, N. , Rosset, S. , Zarate, J. , Ferrari, E. , Brayda, L. , & Shea, H. (2017). Understanding graphics on a scalable latching assistive haptic display using a shape memory polymer membrane. IEEE Transactions on Haptics, 11(1), 30–38. 10.1109/TOH.2017.2767049 29611811

[brb31650-bib-0002] Bestgen, Y. , & Dupont, V. (2003). The construction of spatial situation models during reading. Psychological Research Psychologische Forschung, 67, 209–218. 10.1007/s00426-002-0111-8 12955510

[brb31650-bib-0003] Braun, V. , & Clarke, V. (2013). Teaching thematic analysis: Over‐coming challenges and developing effective strategies for effective learning. The Psychologist, 26(2), 120–123.

[brb31650-bib-0004] Brayda, L. , Leo, F. , Baccelliere, C. , Ferrari, E. , & Vigini, C. (2018). Updated tactile feedback with a pin array matrix helps blind people to reduce self‐location errors. Micromachines, 9(7), 351 10.3390/mi9070351 PMC6082250 30424284

[brb31650-bib-0005] Brock, A. M. , Truillet, P. , Oriola, B. , Picard, D. , & Jouffrais, C. (2015). Interactivity improves usability of geographic maps for visually impaired people. Human‐Computer Interaction, 30(2), 156–194. 10.1080/07370024.2014.924412

[brb31650-bib-0006] Campus, C. , Brayda, L. , De Carli, F. , Chellali, R. , Famà, F. , Bruzzo, C. , … Rodriguez, G. (2012). Tactile exploration of virtual objects for blind and sighted people: The role of beta 1 EEG band in sensory substitution and supramodal mental mapping. Journal of Neurophysiology, 107(10), 2713–2729. 10.1152/jn.00624.2011 22338024 PMC3362272

[brb31650-bib-0007] Cattaneo, Z. , Vecchi, T. , Cornoldi, C. , Mammarella, I. , Bonino, D. , Ricciardi, E. , & Pietrini, P. (2008). Imagery and spatial processes in blindness and visual impairment. Neuroscience & Biobehavioral Reviews, 32, 1346–1360. 10.1016/j.neubiorev.2008.05.002 18571726

[brb31650-bib-0008] Chiesa, S. , Schmidt, S. , Tinti, C. , & Cornoldi, C. (2017). Allocentric and contra‐aligned spatial representations of a town environment in blind people. Acta Psychologica, 180, 8–15. 10.1016/j.actpsy.2017.08.001 28806576

[brb31650-bib-0009] Cohen, N. J. , & Eichenbaum, H. (1991). The theory that wouldn't die: A critical look at the spatial mapping theory of hippocampal function. Hippocampus, 1(3), 265–268. 10.1002/hipo.450010312 1669304

[brb31650-bib-0010] Eden, C. (1988). Cognitive mapping. European Journal of Operational Research, 36, 1–13. 10.1016/0377-2217(88)90002-1

[brb31650-bib-0011] Ertan, S. , Lee, C. , Willets, A. , Tan, H. , & Pentland, A. (1998). A wearable haptic navigational guidance system. Wearable Computers, 1998. Digest of Papers. Second International Symposium.

[brb31650-bib-0012] Field, A. P. (2018). Discovering statistics using IBM SPSS statistics (5th ed.). London, UK: Sage.

[brb31650-bib-0013] Gagnon, L. , Kupers, R. , & Ptito, M. (2013). Reduced taste sensitivity in congenital blindness. Chemical Senses, 38, 509–517. 10.1093/chemse/bjt021 23667250

[brb31650-bib-0014] Geronazzo, M. , Bedin, A. , Brayda, L. , Campus, C. , & Avanzini, F. (2016). Interactive spatial sonification for non‐visual exploration of virtual maps. International Journal of Human‐Computer Studies, 85, 4–15. 10.1016/j.ijhcs.2015.08.004

[brb31650-bib-0015] Goldreich, M. , & Kanics, I. M. (2006). Performance of blind and sighted humans on a tactile grating detection task. Perception & Psychophysics, 68(8), 1363–1371. 10.3758/BF03193735 17378422

[brb31650-bib-0016] Gori, M. , Sandini, G. , Martinoli, C. , & Burr, D. C. (2014). Impairment of auditory spatial localization in congenitally blind human subjects. Brain, 137(1), 288–293. 10.1093/brain/awt311 24271326 PMC3891446

[brb31650-bib-0017] Graf, C. (2010). Verbally annotated tactile maps: Challenges and approaches. Spatial Cognition VII, 6222, 303–318.

[brb31650-bib-0018] Jacobson, R. D. , & Kitchin, R. M. (2012). GIS and people with visual impairments or blindness: Exploring the potential for education, orientation, and navigation. Transactions in GIS, 2(4), 315–332. 10.1111/j.1467-9671.1997.tb00060.x

[brb31650-bib-0019] Johnson‐Laird, P. N. (1989). Mental models In Posner M. I. (Ed.), Foundations of cognitive science. Cambridge, MA: MIT Press.

[brb31650-bib-0020] Katz, B. F. G. , Kammoun, S. , Parseihian, G. , Gutierrez, O. , Brilhault, A. , Auvray, M. , … Jouffrais, C. (2012). NAVIG: Augmented reality guidance system for the visually impaired. Virtual Reality, 16, 253 10.1007/s10055-012-0213-6

[brb31650-bib-0021] Katz, B. F. G. , & Picinali, L. (2011). Spatial audio applied to research with the blind In Sturmillo P. (Ed.), Advances in sound localization (pp. 225‐252). Rijeka, croatia: Europe, INTECH ISBN: 978‐953‐307‐224‐1. Availiable from: http://www.intechopen.com/books/advances-in-sound-localization/spatial-audio-applied-to-research-with-the-blind

[brb31650-bib-0022] Kosslyn, S. M. (1880). Image and mind. Cambridge, MA: Harvard University Press.

[brb31650-bib-0023] Kraska‐Miller, M. (2014). Nonparametric statistics for social and behavioural science. New York, NY: CRC Press.

[brb31650-bib-0024] Kupers, R. , Chebat, D. R. , Madsen, K. H. , Paulson, O. B. , & Ptito, M. (2010). Neural correlates of virtual route recognition in congenital blindness. Proceedings of the National Academy of Sciences of the United States of America, 107(28), 12716–12721. 10.1073/pnas.1006199107 20616025 PMC2906580

[brb31650-bib-0025] Leo, F. , Violin, T. , Inuggi, A. , Raspagliesi, A. , Capris, E. , Cocchi, E. , & Brayda, L. (2019). Blind persons get improved sense of orientation and mobility in large outdoor spaces by means of a tactile pin‐array matrix. Position paper submitted to CHI 2019 Workshop on Hacking Blind Navigation.

[brb31650-bib-0026] Loomis, J. M. , Klatzky, R. L. , Golledge, R. G. , Cicinelli, J. G. , Pellegrino, J. W. , & Fry, P. A. (1993). Nonvisual navigation by blind and sighted: Assessment of path integration ability. Journal of Experimental Psychology: General, 122(1), 73–91. 10.1037/0096-3445.122.1.73 8440978

[brb31650-bib-0027] Marebet, L. B. , Connors, E. , Halko, M. A. , & Sánchez, J. (2012). Teaching the blind to find their way by playing video games. PLoS ONE, 7(9), e44958 10.1371/journal.pone.0044958 23028703 PMC3446956

[brb31650-bib-0028] Martinez‐Sala, A. S. , Losilla, F. , Sánchez‐Aarnoutse, J. C. , & García‐Haro, J. (2015). Design, implementation and evaluation of an indoor navigation system for visually impaired people. Sensors, 15(12), 32168–32187. 10.3390/s151229912 26703610 PMC4721829

[brb31650-bib-0029] Meliones, A. , & Sampson, D. (2018). Blind museum tourer: A system for self‐guided tours in museums and blind indoor navigation. Technologies, 6(1), 4 10.3390/technologies6010004

[brb31650-bib-0030] Noordzij, M. L. , Zuidhoek, S. , & Postma, A. (2006). The influence of visual experience on the ability to form spatial mental models based on route and survey descriptions. Cognition, 100, 321–342. 10.1016/j.cognition.2005.05.006 16043169

[brb31650-bib-0031] O'Keefe, J. , & Nadel, L. (1978). The hippocampus as a cognitive map. Oxford, UK: Oxford University Press.

[brb31650-bib-0032] O'Sullivan, L. , Picinali, L. , Gerino, A. , & Cawthorne, D. (2015). A prototype audio‐tactile map system with an advanced auditory display. International Journal of Mobile Human Computer Interaction, 7, 53–75. 10.4018/IJMHCI.2015100104

[brb31650-bib-0033] Papadopoulos, K. , Koustriava, E. , & Koukourikos, P. (2018). Orientation and mobility aids for individuals with blindness: Verbal description vs. audio‐tactile map. Assistive Technology, 30(4), 1–10. 28471302 10.1080/10400435.2017.1307879

[brb31650-bib-0034] Picinali, L. , Afonso, A. , Denis, M. , & Katz, B. F. G. (2014). Exploration of architectural spaces by blind people using auditory virtual reality for the construction of spatial knowledge. International Journal of Human Computer Studies, 72(4), 393–407.

[brb31650-bib-0035] Roder, B. , der Sälejärvi, W. , Sterr, A. , Rösler, F. , Hillyard, S. A. , & Neville, H. J. (1999). Improved auditory spatial tuning in blind humans. Nature, 400, 162–166. 10.1038/22106 10408442

[brb31650-bib-0036] Roder, B. , & Neville, H. (2003). Developmental functional plasticity In Grafman S., & Robertson I. H. (Eds.), Handbook of neuropsychology (vol. 9, 2nd ed., pp. 231–270). Amsterdam, The Netherlands: Elsevier.

[brb31650-bib-0037] Roentgen, U. R. , Gelderblom, G. J. , Soede, M. , & de Witte, L. P. (2008). Inventory of electronic mobility aids for persons with visual impairments: A literature review. Journal of Visual Impairment & Blindness, 102(11), 702–724. 10.1177/0145482X0810201105

[brb31650-bib-0038] Strelow, E. R. (1985). What is needed for a theory of mobility: Direct perceptions and cognitive maps‐lessons from the blind. Psychological Review, 92(2), 226–248. 10.1037/0033-295X.92.2.226 3887451

[brb31650-bib-0039] Teddlie, C. , & Tashakkori, A. (2009). Foundations of mixed methods research: Integrating quantitative and qualitative approaches in the social and behavioral sciences. Thousand Oaks, CA: Sage.

[brb31650-bib-0040] Tolman, E. C. (1948). Cognitive maps in rats and men. The Psychological Review, 55(4), 189–208. 10.1037/h0061626 18870876

[brb31650-bib-0041] Tversky, B. (1991). Spatial mental models In Bower G. H. (Ed.), The psychology of learning and motivation: Advances in research and theory. San Diego, CA: Academic Press Inc.

[brb31650-bib-0042] Ungar, S. , Blades, M. , & Spencer, C. (1993). The role of tactile maps in mobility training. British Journal of Visual Impairment, 11(2), 59–61. 10.1177/026461969301100205

[brb31650-bib-0043] Velázquez, R. , Pissaloux, E. , Rodrigo, P. , Carrasco, M. , Carrasco, M. , & Giannoccaro, N. I. (2018). An outdoor navigation system for blind pedestrians using GPS and tactile‐foot feedback. Applied Sciences, 8(4), 578 10.3390/app8040578

[brb31650-bib-0044] Zeng, L. , & Weber, G. (2011). AT Map: Annotated tactile maps for the visually impaired In Esposito A., Esposito A. M., Vinciarelli A., Hoffmann R., & Müller V. C. (Eds.), Cognitive Behavioural Systems, LNCS, 7403 (pp. 290–298). Heidelberg: Springer.

